# Factors Associated with the Digital Patient Experience of Virtual Care Across Specialties

**DOI:** 10.1089/tmr.2023.0032

**Published:** 2023-08-03

**Authors:** Kori S. Zachrison, Zhiyu Yan, Benjamin A. White, Lee Park, Lee H. Schwamm

**Affiliations:** ^1^ Department of Emergency Medicine, Massachusetts General Hospital, Boston, Massachusetts, USA.; ^2^ Department of Internal Medicine, Brigham & Women's Hospital, Boston, Massachusetts, USA.; ^3^ Mass General Brigham, Boston, Massachusetts, USA.; ^4^ Department of Neurology, Massachusetts General Hospital, Boston, Massachusetts, USA.

**Keywords:** telehealth, ambulatory care, patient experience

## Abstract

**Background:**

We aimed to characterize patient experience with virtual care across medical specialties using validated survey data. Primary objective: to determine whether experience varied by visit modality (virtual vs. in-person) and whether relationships persisted after adjusting for patient and provider characteristics. Secondarily, among physicians with sufficient data, we compared virtual versus in-person patient experience scores at the physician level and identified characteristics associated with better experience scores for virtual care.

**Methods:**

This was a retrospective analysis of administrative databases from a large New England health care system, including all ambulatory visits from October 1, 2020 to September 30, 2021 with patient experience scores recorded. We compared experience between virtual and in-person at the visit level (score: 0–10) and the physician level for likelihood of recommending the physician to friends or family. We used a series of cross-classified hierarchical models with visits grouped by patient and by physician to decompose sources of variation. Among physicians with sufficient data, we compared physicians with higher virtual versus higher in-person net promoter score (NPS).

**Results:**

Of 378,472 visits performed by 3368 physicians, 86,878 (23%) were conducted virtually. Most scored ≥9 for either modality, with a small preference for virtual versus in-person care (9.6 vs. 9.5, *p* < 0.001). We found that more variation in scores was explained by patient than by physician (22.9% vs. 3%). Visit modality was of minimal explanatory value. Most physicians' virtual and in-person NPS were similar, and virtual visit volume was not associated.

**Conclusions:**

We found robust evidence for the parity of patient experience between virtual and in-person modalities across specialties.

## Introduction

Virtual care has become a fixture in the U.S. health care delivery system. Growth has been exponential during the pandemic, particularly during variant surges and among behavioral health visits.^1,2^

Studies have demonstrated that in general, patients are interested in receiving health care virtually. Patients have indicated that they are satisfied with the care received and its convenience.^1,3–7^ The majority patients participating in a community survey in Australia noted that their experience with virtual care was as good as or better than traditional in-person care.^8^ However, a recent survey of over 2000 U.S. patients found that when faced with the choice, the majority of patients preferred to have an in-person visit over a virtual visit.^9^ Outside of a small number of specialty-specific studies,^10–13^ this question has not yet been addressed using data directly from patients' experience associated with specific ambulatory visit encounters.

Therefore, using validated patient experience survey data collected in a national leading vendor platform, we sought to determine whether patient experience scores varied by visit modality (virtual vs. in-person) and to what extent these relationships persisted within hierarchical models adjusted for patient and provider characteristics. Secondarily, among physicians with sufficient data, we compared virtual versus in-person patient experience scores at the physician level and identified characteristics associated with better experience scores for virtual care.

## Methods

### Setting, population, and sources of data

This study was conducted using data from a large regional health care system that includes outpatient practice sites affiliated with 12 academic and community hospitals and was approved by our organizational IRB. We used enterprise-wide electronic health record data (Epic Systems) to identify ambulatory visits and the characteristics of patients having the visits. We used systemwide data on patient experience during ambulatory visits collected by National Research Corporation Health (Lincoln, NE). Finally, we used data from our organizational master provider credentialing database to extract relevant physician characteristics.

We identified all ambulatory patient encounters during the October 1, 2020–September 30, 2021 study period. Visits were categorized as virtual versus in-person. Virtual visits included both video and audio only. We included all visits that had patient experience data with a recorded response for “Would you recommend this physician to your friends and family?” Because we were interested in identifying differences between practice types, we excluded visits without identifiable physician practice designation.

From the overall sample, we also identified a subset of visits to address our secondary objective. We identified physicians who had at least 10 in-person and 10 virtually conducted ambulatory visits with recorded scores for “Would you recommend this physician to your friends and family?” This sample was designated as our within-physician comparison sample.

### Outcomes of interest

Our primary outcome was at the visit level, based on patients' response to the question “Would you recommend this physician to your friends and family?” Response options range from 0 to 10 and we used the raw scores for analysis and interpretation.

At the physician level, we analyzed the net promoter score (NPS), the standard approach to evaluating patient experience or satisfaction with their physician's performance, calculated as the proportion of scores considered promoters (9 or 10) out of all scored visits minus the proportion of scores considered detractors (0 through 6) out of all scored visits. Passive scores (7 and 8) are not included in the calculation. We calculated an overall NPS for each physician based on all of their visits, as well as in-person- and virtual-specific NPS using only in-person and only virtual visits, respectively.

### Other variables of interest

At the visit level, we characterized visits by patient age, gender, race, ethnicity, language preference, insurance status, and electronic health portal activation. Visits were also categorized as belonging to primary care, medical specialty, surgical specialty, or behavioral health based on the primary specialty of the physician performing the visit.

We characterized physicians by age and popularized generational demographic cohort (Silent Generation: 1928–1945; Baby Boomers: 1946–1964; Generation X: 1965–1980; Millennials: 1981–1996) using the Brookings Institute classification.^14^ We also characterized physicians by gender, years since medical school graduation, and academic practice, dichotomously based on affiliation with at least one of the major teaching hospitals in our system. We also created a set of physician level attributes derived from characteristics of the patients they treated during the study period. This included the number of unique patients with whom they had any type of visit during the study period, and the proportion of patients with self-pay or Medicaid insurance, who were aged 65 years or older, who prefer speaking a language other than English, who self-report belonging to a racial or ethnic minority group, and who have an active account in our electronic health patient portal (Epic MyChart).

### Statistical analysis

We used standard descriptive statistics to characterize the sample of visits overall and by virtual versus in-person visit modality. We used standardized mean differences (SMD) to identify meaningful differences between groups and considered an SMD greater than 0.1 as a meaningful difference. We used Kendall Tau-b nonparametric testing to compare raw patient experience scores between virtual and in-person visits overall and by specialty and chi-square testing to compare top box versus non-top box scores for virtual and in-person visits. We used linear regression models to examine trends over time in visit scores overall, by visit modality, and by specialty type. We then used a series of mixed models with cross-level random effects to decompose sources of variation in patient experience scores, with encounters grouped by patients and physicians.

We examined changes in the intraclass correlation coefficient at the patient and physician levels. The first model (Model 0) was unadjusted, including only random intercepts for patient and physician. Model 1 added visit modality (virtual vs. in-person). Model 2 additionally included physician specialty (primary care, medical specialty, surgical specialty, behavioral health). Model 3 added patient case-mix to Model 2 (age, gender, race, ethnicity, language preference, Medicaid status, and portal activation status). Model 4 added physician characteristics to Model 3 (age, gender, years since medical school graduation, affiliation with major teaching hospital, number of unique patients, proportion of visits by Medicaid patients, proportion of visits by patients ≥65 years of age, proportion of visits by non-English preferring patients, proportion of visits by patients with activated portal, and proportion of visits by nonwhite patients).

We next identified the physicians with sufficient patient experience data for both in-person and virtual visits and compared characteristic physicians included versus those excluded from this component of the analysis using *t*-tests and SMD. Among included physicians, we compared NPS scores virtually versus in-person overall using ordinary least squares (OLS) regression, and we compared individual physicians' virtual versus in-person NPS using paired *t*-tests and SMD. We also calculated correlation coefficients between physicians' in-person and virtual NPSs overall and by specialty group.

Finally, for each physician, we calculated the numerical ratio of their virtual versus in-person NPSs. We examined the distribution of these ratios overall and by specialty type and used these ratios to identify physicians in the highest quartile (stronger virtually) versus the lowest quartile (stronger in-person). We used *t*-tests and SMD to characterize differences between physicians with stronger virtual versus strong in-person patient experience.

## Results

**FIG. 1.**
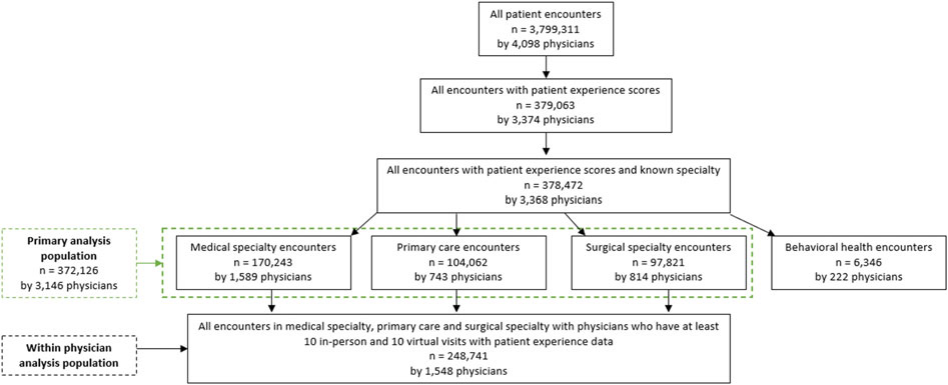
Patient inclusion flow diagram.

Of the 3,799,311 ambulatory in-person and virtual visits conducted by 4098 of our physicians during the October 2020–September 2021 study period, 378,472 visits had associated patient experience data recorded with known physician specialty. Of these, 170,243 (45%) were medical specialty visits, 104,063 (27%) were primary care visits, 97,821 (26%) were surgical specialty visits, and 6346 (2%) were behavioral health visits (Fig. 1). Because the number of behavioral health visits conducted in-person was very small (290, or <0.1% of all visits), behavioral health visits were included in our global analysis, but excluded from those analyses comparing virtual and in-person patient experience.

### Encounter characteristics

Of the 378,472 visits in our analytic sample, 86,878 (23%) were conducted virtually and 291,594 (77%) in-person. Of all ambulatory visits, 170,243 (45%) were medical specialty visits (47,445 or 28% of which were virtual); 104,062 (28%) were primary care (17,793 or 17% of which were virtual); 97,821 (26%) were surgical specialty visits (15,584 or 16% of which were virtual); and 6346 (2%) were behavioral health (6056 or 95% of which were virtual). Median age was 66 years (interquartile range 54–75) and 60% of visits were by female patients. Gender, race, and ethnicity were similar between in-person and virtual visits (Table 1).

**Table 1.** Encounter Characteristics

	Overall (***N*** = 378,472)	In-person (***N*** = 291,594)	Virtual (***N*** = 86,878)	Standardized mean difference
Characteristics of patients in encounters				
Age				
Mean (SD)	62.2 (18.1)	62.8 (18.0)	60.0 (18.2)	0.16
Gender				
Female	225,910 (59.7%)	172,907 (59.3%)	53,003 (61.0%)	0.04
Race				
White	319,320 (84.4%)	245,431 (84.2%)	73,889 (85.0%)	0.02
Black	17,226 (4.6%)	13,383 (4.6%)	3843 (4.4%)	
Asian	12,639 (3.3%)	9605 (3.3%)	3034 (3.5%)	
Other	15,014 (4.0%)	11,806 (4.0%)	3208 (3.7%)	
Missing	14,273 (3.8%)	11,369 (3.9%)	2904 (3.3%)	
Ethnicity				
Non-Hispanic	324,593 (85.8%)	249,204 (85.5%)	75,389 (86.8%)	0.02
Hispanic	19,543 (5.2%)	15,273 (5.2%)	4270 (4.9%)	
Missing	34,336 (9.1%)	27,117 (9.3%)	7219 (8.3%)	
Language				
English	360,029 (95.1%)	276,283 (94.7%)	83,746 (96.4%)	0.08
Not English	18,369 (4.9%)	15,253 (5.2%)	3116 (3.6%)	
Missing	74 (0.0%)	58 (0.0%)	16 (0.0%)	
Medicaid				
Non-Medicaid	353,923 (93.5%)	272,753 (93.5%)	81,170 (93.4%)	0.00
Medicaid	20,144 (5.3%)	15,478 (5.3%)	4666 (5.4%)	
Missing	4405 (1.2%)	3363 (1.2%)	1042 (1.2%)	
Portal				
Activated	323,769 (85.5%)	242,004 (83.0%)	81,765 (94.1%)	0.35
Not activated	52,452 (13.9%)	47,548 (16.3%)	4904 (5.6%)	
Missing	2251 (0.6%)	2042 (0.7%)	209 (0.2%)	
Characteristics of physicians performing encounters				
Age				
Mean (SD)	50.8 (11.4)	50.9 (11.4)	50.5 (11.4)	0.04
Missing	4454 (1.2%)	3835 (1.3%)	619 (0.7%)	
Generation				
Silent Generation (1928–1945)	7837 (2.1%)	5431 (1.9%)	2406 (2.8%)	0.11
Boomers (1946–1964)	113,243 (29.9%)	90,083 (30.9%)	23,160 (26.7%)	
Gen X (1965–1980)	176,975 (46.8%)	134,643 (46.2%)	42,332 (48.7%)	
Millennials (1981–1996)	75,963 (20.1%)	57,602 (19.8%)	18,361 (21.1%)	
Missing	4454 (1.2%)	3835 (1.3%)	619 (0.7%)	
Gender				
Female	168,366 (44.5%)	129,097 (44.3%)	39,269 (45.2%)	0.01
Missing	4454 (1.2%)	3835 (1.3%)	619 (0.7%)	
Years since medical school graduation				
Mean (SD)	23.6 (11.8)	23.6 (11.8)	23.6 (11.7)	<0.01
Missing	51,656 (13.6%)	45,435 (15.6%)	6221 (7.2%)	
Major teaching hospital affiliation				
Yes	284,628 (75.2%)	210,844 (72.3%)	73,784 (84.9%)	0.31
Specialty				
Medical	170,243 (45.0%)	122,798 (42.1%)	47,445 (54.6%)	0.51
Primary care	104,062 (27.5%)	86,269 (29.6%)	17,793 (20.5%)	
Surgical	97,821 (25.8%)	82,237 (28.2%)	15,584 (17.9%)	
Behavioral health	6346 (1.7%)	290 (0.1%)	6056 (7.0%)	
No. of unique patients seen during study year				
Mean (SD)	944 (595)	1020 (611)	692 (452)	0.61
Proportion of patients self-pay or Medicaid				
Mean (SD)	8.95 (8.95)	8.81 (9.16)	9.39 (8.16)	0.07
Proportion of patients 65 years and older				
Mean (SD)	40.0 (19.3)	40.3 (18.8)	38.8 (20.7)	0.08
Proportion of patients non-English preferring				
Mean (SD)	6.37 (7.21)	6.42 (7.30)	6.20 (6.90)	0.03
Median (Q1–Q3)	4.76 (2.64–7.49)	4.71 (2.60–7.55)	4.85 (2.84–7.34)	
Proportion of patients with activated portal				
Mean (SD)	83.4 (12.0)	82.0 (12.5)	88.1 (8.67)	0.57
Proportion of patients nonwhite				
Mean (SD)	19.1 (12.8)	19.0 (13.1)	19.5 (11.9)	0.04

SD, standard deviation.

At the encounter-level, virtual visits were more frequently performed by physicians who were from younger generations, associated with one of the major academic hospitals in the system, practicing in a medical specialty or behavioral health, with a smaller number of patients seen in the study period, and a higher proportion of patients with activated patient portals.

### Patient experience

**FIG. 2.**
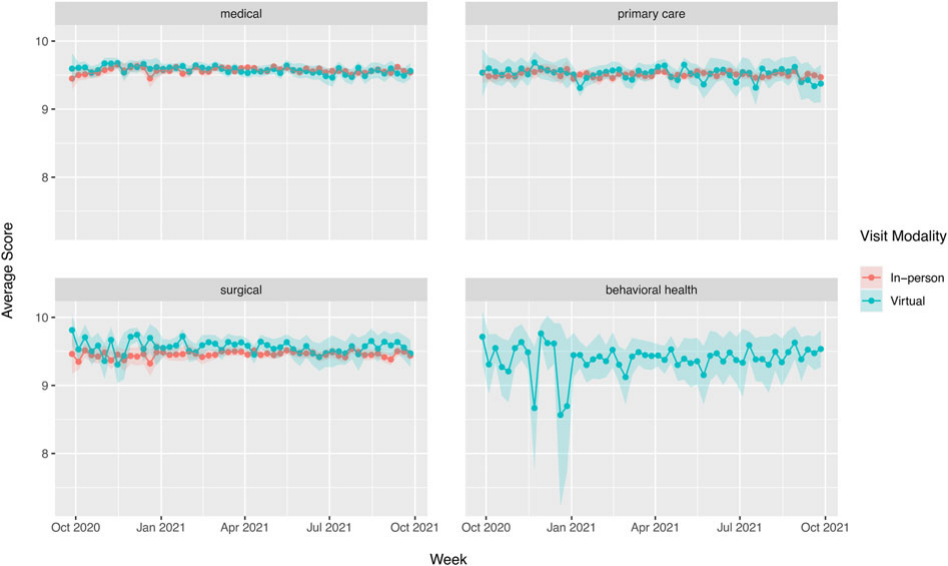
Proportion of visits receiving top box score, by specialty and visit type. The *shaded areas* represent the 95% Wald confidence intervals for the proportion.

The majority of encounters in all specialties, virtual and in-person, received scores of 9 or 10 (i.e., “top box scores”) on the 10-point scale (medical specialties: in-person 91.9%, virtual 91.7%; primary care: in-person 90.4%, virtual 90.3%; surgical specialties; in-person 89.8%, virtual 91.3%; behavioral health: in-person 89.3%, virtual 88.5%). The proportion of visits receiving a top box score was stable over time (Fig. 2).

Overall, raw score for “Would you recommend this physician to your friends or family” for virtual visits were statistically higher than for in-person visits (9.5 vs. 9.6, *p* < 0.001) but it is unclear if this is a clinically meaningful difference. While scores for in-person visits had a small negative deflection during the winter of 2021, scores were overall stable over the 1-year study period; in contrast virtual encounters had a slight decreasing trend (−0.002 points per week, *p* < 0.001). This was primarily due to a decreasing trend among virtual behavioral health visits (−0.29 points per week, *p* < 0.001); virtual visits among medical specialties, primary care, and surgical specialties had stable trend lines over the study period.

### Sources of variation in patient experience

We used a series of cross-classified three-level models to decompose sources of variation in patient experience scores, with encounters grouped by patients and by physicians. In the unadjusted model, we found that 22.9% of variation in patient experience scores was explained by patient differences, and 3% was explained by physician differences. Visit modality added little explanatory value. After fully adjusting for visit modality (virtual vs. in-person), specialty type, patient case-mix, and physician characteristics, only a small amount of additional variation in patient experience scores was explained. In the fully adjusted model, 21.1% of variance in scores remained at the patient-level and 2.5% at the physician level (Supplementary Table S1).

### Physician-level comparison of virtual versus in-person patient experience

We were next interested in understanding if there would be significant differences in how patients experience the same provider through virtual versus in-person visits. In other words, do patients rate physicians differently in these environments, which might reflect differences in behavior or communication in these two environments. Of the 4098 physicians in our overall sample, 1548 (38%) had sufficient data with at least 10 scored visits for both virtual and in-person patient experience. Characteristics of physicians included versus excluded from this analysis are detailed in Supplementary Table S2.

**FIG. 3.**
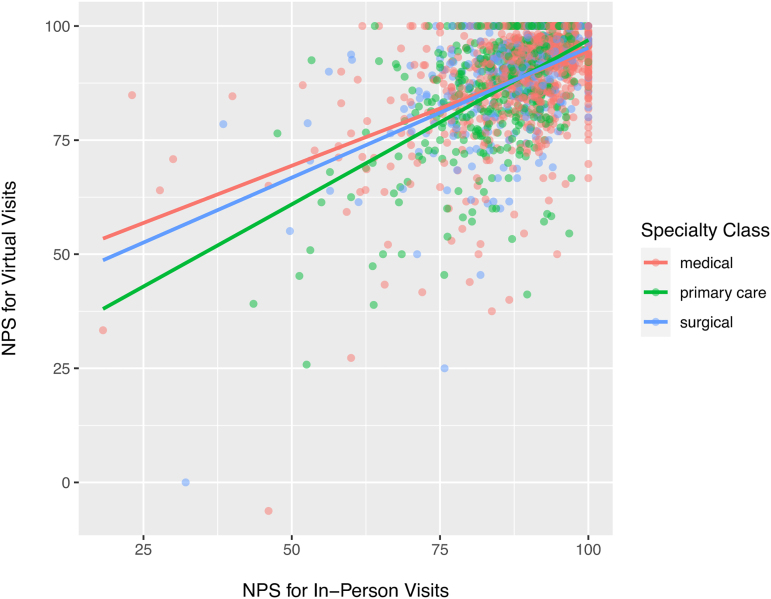
Scatterplot of virtual × in-person net promoter scores, by physician specialty. NPS, net promoter score.

We first calculated three NPS for each physician, an overall score, a score for their virtual visits only and a score for their in-person visits only. Overall, the physician-level mean that NPSs for virtual versus in-person visits were similarly high (virtual 88.1, in-person 87.6; SMD for difference 0.04). In a paired *t*-test examining virtual versus in-person scores within physicians, there was no significant difference between physicians' virtual and in-person NPSs (95% confidence interval for difference −0.09 to 1.0). Physicians' virtual and in-person NPS demonstrated relatively strong, positive correlation (Fig. 3; correlation coefficient 0.49 overall; among physicians in medical specialties: 0.47, in primary care: 0.53, and in surgical specialties: 0.49).

For each physician, we calculated the ratio of their virtual-versus-in-person NPSs, so that a ratio of 1.0 would indicate no difference in virtual versus in-person scores, >1 would indicate higher virtual NPS and <1 indicating higher in-person NPS. The median ratio overall was 1.01 (among medical specialties: 1.01, primary care: 1.01, and surgical specialties 1.02) and 90% of physicians had a ratio between 0.80 and 1.22. The distributions of physician virtual-versus-in-person ratios stratified by specialty, are illustrated in the Supplementary Fig. S1. Relative to physicians with ratios in the bottom quartile (stronger in-person), physicians in the top quartile (stronger virtually) were more often Millennials, less often female, more often primary care and surgical specialties and had higher proportion of patients on Medicaid or self-pay, non-English preferring, and patients of nonwhite race/ethnicity (Table 2). There was no difference between groups in the proportion of visits conducted virtually.

**Table 2.** Comparison of Physicians with Stronger Virtual Versus Stronger In-Person Patient Experience

	Quartile with performance favoring in-person, ***n*** = 387	Quartile with performance favoring virtual, ***n*** = 387	Standardized mean difference
Age, mean (SD)	49.75 (10.98)	48.80 (10.87)	0.09
Generation (%)			
Silent Generation (1928–1945)	4 (1.0)	4 (1.0)	0.10
Boomers (1946–1964)	106 (27.7)	100 (26.2)	
Gen X (1965–1980)	187 (49.0)	176 (46.1)	
Millennials (1981–1996)	85 (22.3)	102 (26.7)	
Female, *n* (%)	198 (51.8)	176 (46.1)	0.12
Years since medical school graduation, mean (SD)	22.81 (11.37)	21.79 (11.34)	0.09
Affiliated with major teaching hospital, *n* (%)	323 (83.5)	335 (86.6)	0.09
Specialty, *n* (%)			
Medical	206 (53.2)	162 (41.9)	0.24
Primary care	115 (29.7)	133 (34.4)	
Surgical	66 (17.1)	92 (23.8)	
No. of patients, mean (SD)	684.3 (443.3)	673.6 (459.2)	0.02
Proportion of patients self-pay or Medicaid, mean (SD)	9.5 (7.7)	11.1 (10.4)	0.17
Proportion of patients 65 years and older, mean (SD)	37.2 (21.0)	34.2 (19.8)	0.15
Proportion of patients non-English preferring, mean (SD)	6.5 (6.6)	7.4 (8.6)	0.11
Proportion of patients with activated patient portal, mean (SD)	86.5 (9.5)	86.3 (10.2)	0.02
Proportion of patients nonwhite, mean (SD)	21.5 (12.9)	23.2 (15.1)	0.13
Proportion of patient visits conducted virtually, mean (SD)	39.0 (23.5)	39.1 (22.3)	<0.01

## Discussion

In this comprehensive study of patient experience in a large integrated academic health system, we found that patient experience scores overall for ambulatory appointments with physicians did not differ by visit modality between virtual versus in-person visits. This held true not only in the overall aggregate analysis but also even when analyzed at the individual physician level. Most physicians who conducted both in-person and virtual visits had similar NPS regardless of visit modality. Thus, our findings suggest that it is the care that is rendered or the relationship with the provider, rather than visit modality, that determines the NPS rating of patient experience.

Our finding that patient experience scores for virtual and in-person care were extremely high and relatively stable over the year-long study period is similar to prior work that has documented high patient satisfaction with virtual visits.^5,15^ Our results add to the prior literature with newer more comprehensive data, comparison across different specialties, and identification of sources of variation in patient experience scores. We found that patient experience scores across different specialties were high and were sustained over time, with only a slight decreasing trend over time among virtual behavioral health visits of uncertain clinical significance. Of all the variation in patient experience NPS, only 3% was explained by differences at the physician level. In summary and perhaps contrary to expectation, variation in patient experience was minimally explained by visit modality, patient case-mix, or physician characteristics.

We believe that we are also one of the first to report within physician differences between virtual versus in-person patient experience and we found that overall, scores were not significantly different by virtual versus in-person visit modality. We did find some variation in the ratio of in-person versus virtual NPS, with virtual performance better in some physicians and worse in others, although these differences were quite small. Interestingly, this did not seem to be related to a physician familiarity factor with virtual care, as the proportion of visits conducted virtually by physicians with better virtual NPS was similar to those that had better in-person NPS.

Our findings help to better illustrate the drivers of patient experience within virtual care models. For example, it remains an open question as to whether patient satisfaction with the virtual experience varies based on availability of in-person care; that is, do scores go up when no alternative option of being seen in-person is available? Given the naturally occurring variation in availability of in-person care during the study period and the stability of patient experience NPS over time, our results suggest that availability of in-person care did not influence patients' rating of virtual care. In addition, it is possible that patient experience with virtual care might depend on physicians' virtual visit volume and competency, and how frequently they use virtual platforms for care delivery.

Our findings suggest otherwise, with minimal differences evident in the data. Our study also suggests that known contributors to excellent patient experience, specifically listening to patients, conveying empathy, and communicating well, remain important factors whether a visit is virtual or in-person.^16^ While we were not able to assess such relationship-centered measures in this dataset, other research has suggested that relational experiences during a virtual visit are an important component of the virtual patient experience and that patient-clinician engagement and relationship-centered measures were similar between virtual and in-person visits.^15,17^ It is important to note that there is some debate as to the value and reliability of patient experience data as currently incorporated into the health care system and physician incentives.^18,19^

Our findings are also limited as they represent those of a single academic integrated delivery network health system in New England in a period, including the COVID-19 pandemic and thus generalizability to other practice settings, regions, or time periods is unclear. In addition, we were only able to collect patient experience data from patients who successfully completed visits. This may introduce selection bias toward a better virtual patient experience by limiting those who had trouble with technical or connectivity issues. However, this limitation is somewhat offset by the inclusion of audio-only visits. In our anecdotal experience, physicians in our health system who encounter technical difficulty connecting by video with patients often convert to an audio visit rather than cancelling outright.

Finally, the outcome that we used in this study was related to the patient's experience with the *physician.* It is possible that patients could have had a poor experience with a virtual visit but still rated their physician highly; however, patient experience remained strong when we examined behavioral health visits, a specialty in which almost all visits are conducted virtually. In future work, it will be valuable to examine additional components of the patient experience, including specifically related to virtual care delivery.

## Conclusions

Our findings demonstrate highly positive patient experience with virtual ambulatory care delivery. We found modest differences at most between physicians in the patient experience of ambulatory care visits, and these differences were not explained by virtual versus in-person visit modality. These findings provide robust evidence for the parity of patient experience between virtual and in-person modalities across health care specialties and over time.

## Supplementary Material

Supplemental data

Supplemental data

Supplemental data

## Abbreviations Used

NPS: net promoter score
SD: standard deviation
SMD: standardized mean differences
