# Supplementary material for: Factors Associated with the Digital Patient Experience of Virtual Care Across Specialties
Source: Telemed Rep. 2023 Aug 3;4(1):227–35. doi: 10.1089/tmr.2023.0032 (PMC10457601; doi:10.1089/tmr.2023.0032)
Supplement: Supplemental data [file Supp_TableS1.docx]

**Supplementary Table 1. Sources of Variation in Patient Experience Scores**

| Model | Specification | Patient level ICC | Physician level ICC | Interpretation |
| --- | --- | --- | --- | --- |
| 0 | Random intercepts for patient and physician | 22.9% | 3.0% | In the unadjusted model, 22.9% of variation in scores is explained by the patient, and 3% by physician |
| 1 | Model 0 +  visit virtual vs. in-person | 22.9% | 2.9% | After adjusting for virtual vs. in-person visit modality, 22.9% of variation in scores is explained by the patient and 2.9% by the physician; 0.1% of the physician-level variation in scores was due to visit modality. |
| 2 | Model 1 +  Physician specialty | 23.0% | 2.9% | Additionally adjusting for physician specialty does not explain additional variance in patient experience score. |
| 3 | Model 2 +  patient case mix | 22.0% | 2.7% | Accounting for patient-case mix explains additional variance in patient experience scores, with 22% remaining at the patient level and 2.7% remaining at the physician level. |
| 4 | Model 3 +  physician characteristics | 21.1% | 2.5% | Accounting for physician characteristics explains additional variance in patient experience scores, with 21.1% remaining at the patient level and 2.5% remaining at the physician level. |
