# Supplementary material for: Factors Associated with the Digital Patient Experience of Virtual Care Across Specialties
Source: Telemed Rep. 2023 Aug 3;4(1):227–35. doi: 10.1089/tmr.2023.0032 (PMC10457601; doi:10.1089/tmr.2023.0032)
Supplement: Supplemental data [file Supp_TableS2.docx]

**Supplementary Table 2. Comparison of Physicians Included vs. Excluded from Within Physician Analysis**

|  | **Overall** n=4098 | **Included** n=1548 | **Excluded** n=2550 | **SMD** |
| --- | --- | --- | --- | --- |
| **Age** |  |  |  |  |
| Mean (SD) | 49.1 (12.1) | 49.8 (11.1) | 48.6 (12.6) | 0.10 |
| Median (Q1-Q3) | 48.0 (39.0-58.0) | 48.0 (41.0-58.0) | 47.0 (38.0-58.0) |  |
| Missing | 178 (4.3%) | 15 (1.0%) | 163 (6.4%) |  |
| **Generation** |  |  |  | 0.19 |
| Silent Gen (1928 - 1945) | 83 (2.0%) | 26 (1.7%) | 57 (2.2%) |  |
| Boomers (1946 - 1964) | 1031 (25.2%) | 416 (26.9%) | 615 (24.1%) |  |
| Gen X (1965 - 1980) | 1718 (41.9%) | 735 (47.5%) | 983 (38.5%) |  |
| Millennials (1981 - 1996) | 1088 (26.5%) | 356 (23.0%) | 732 (28.7%) |  |
| Missing | 178 (4.3%) | 15 (1.0%) | 163 (6.4%) |  |
| **Gender** |  |  |  | 0.03 |
| M | 2075 (50.6%) | 796 (51.4%) | 1279 (50.2%) |  |
| F | 1845 (45.0%) | 737 (47.6%) | 1108 (43.5%) |  |
| Missing | 178 (4.3%) | 15 (1.0%) | 163 (6.4%) |  |
| **Years since medical school graduation** |  |  |  | 0.14 |
| Mean (SD) | 21.8 (12.4) | 22.8 (11.5) | 21.1 (13.0) |  |
| Median (Q1-Q3) | 20.0 (11.0-31.0) | 21.0 (13.0-31.0) | 18.0 (10.0-31.0) |  |
| Missing | 693 (16.9%) | 158 (10.2%) | 535 (21.0%) |  |
| **Major teaching hospital affiliation** |  |  |  | 0.41 |
| No | 1095 (26.7%) | 247 (16.0%) | 848 (33.3%) |  |
| Yes | 3003 (73.3%) | 1301 (84.0%) | 1702 (66.7%) |  |
| **Specialty** |  |  |  | 0.60 |
| behavioral health | 322 (7.9%) | 0 (0%) | 322 (12.6%) |  |
| medical | 1889 (46.1%) | 785 (50.7%) | 1104 (43.3%) |  |
| primary care | 930 (22.7%) | 461 (29.8%) | 469 (18.4%) |  |
| surgical | 929 (22.7%) | 302 (19.5%) | 627 (24.6%) |  |
| Missing | 28 (0.7%) | 0 (0%) | 28 (1.1%) |  |
| **Number of unique patients seen during study year** |  |  |  | 0.46 |
| Mean (SD) | 516 (547) | 665 (435) | 426 (586) |  |
| Median (Q1-Q3) | 337 (101-784) | 577 (326-896) | 160 (43.3-621) |  |
| **Proportion of patients with self-pay or Medicaid insurance** |  |  |  | 0.38 |
| Mean (SD) | 12.4 (13.3) | 9.51 (8.51) | 14.2 (15.3) |  |
| Median (Q1-Q3) | 8.71 (4.92-14.5) | 7.32 (4.57-10.9) | 9.76 (5.35-17.1) |  |
| **Proportion of patients 65 years of age and older** |  |  |  | 0.25 |
| Mean (SD) | 35.0 (23.0) | 38.4 (20.2) | 32.9 (24.4) |  |
| Median (Q1-Q3) | 34.5 (17.7-50.4) | 38.0 (23.8-52.6) | 32.3 (12.5-50.0) |  |
| **Proportion of patients non-English preferring** |  |  |  | 0.21 |
| Mean (SD) | 7.77 (10.1) | 6.51 (6.84) | 8.54 (11.6) |  |
| Median (Q1-Q3) | 5.34 (2.67-8.96) | 5.12 (2.92-7.81) | 5.56 (2.44-10.0) |  |
| **Proportion of patients with activated portal** |  |  |  | 0.56 |
| Mean (SD) | 81.9 (15.5) | 86.8 (9.18) | 78.9 (17.7) |  |
| Median (Q1-Q3) | 86.2 (75.6-92.2) | 89.0 (83.2-93.0) | 83.3 (70.1-91.3) |  |
| **Proportion of non-white patients** |  |  |  | 0.16 |
| Mean (SD) | 22.3 (16.5) | 20.7 (12.9) | 23.3 (18.3) |  |
| Median (Q1-Q3) | 18.3 (12.1-27.7) | 18.0 (12.4-25.2) | 18.6 (11.9-29.2) |  |
| **Proportion of visits conducted virtually** |  |  |  | 0.24 |
| Mean (SD) | 35.0 (32.2) | 39.4 (22.0) | 32.2 (36.8) |  |
| Median (Q1-Q3) | 25.2 (7.21-55.4) | 36.4 (21.1-54.8) | 15.0 (2.30-57.0) |  |
