# Supplementary material for: Factors Associated with the Digital Patient Experience of Virtual Care Across Specialties
Source: Telemed Rep. 2023 Aug 3;4(1):227–35. doi: 10.1089/tmr.2023.0032 (PMC10457601; doi:10.1089/tmr.2023.0032)
Supplement: Supplemental data [file Supp_FigS1.docx]

**Supplementary Figure. Distribution of Physicians’ Virtual to In-person Net Promoter Score Ratio, by Specialty**

| Medical Specialty  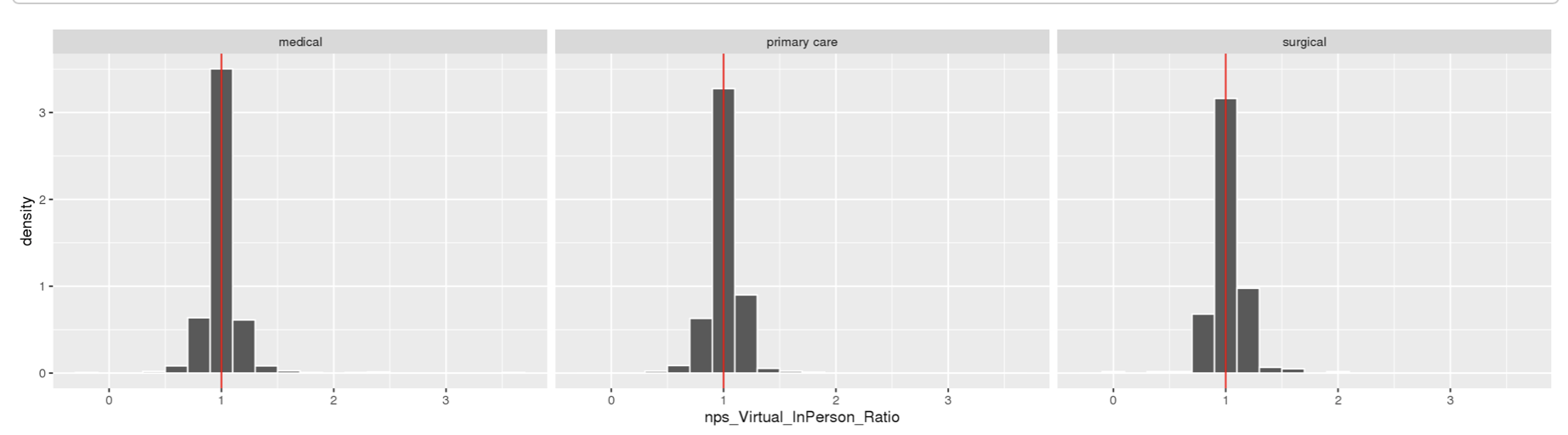 | Primary Care  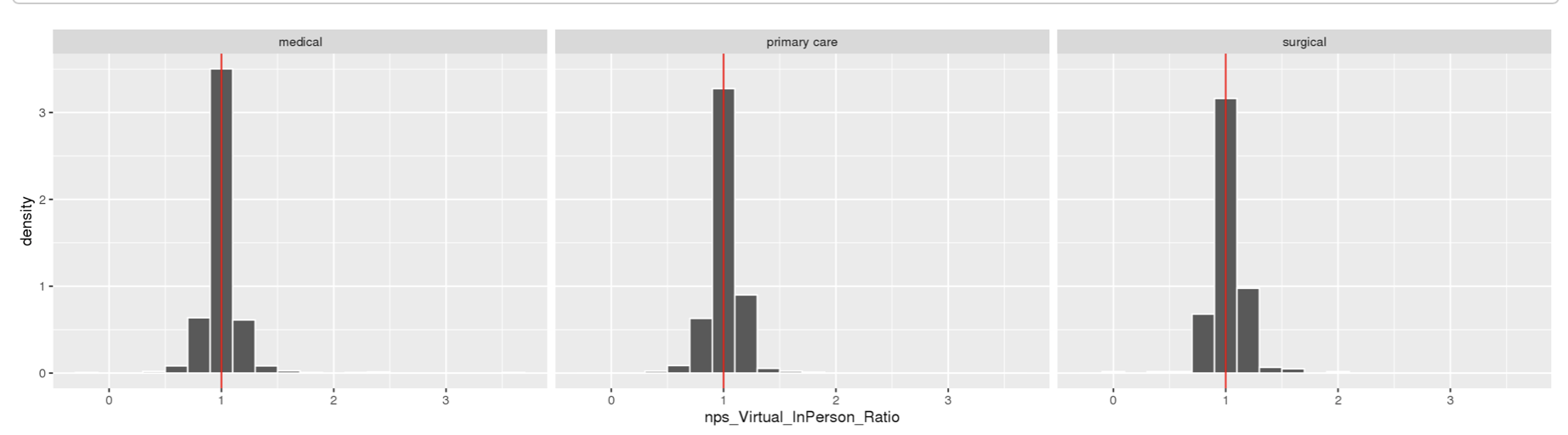 | Surgical Specialty  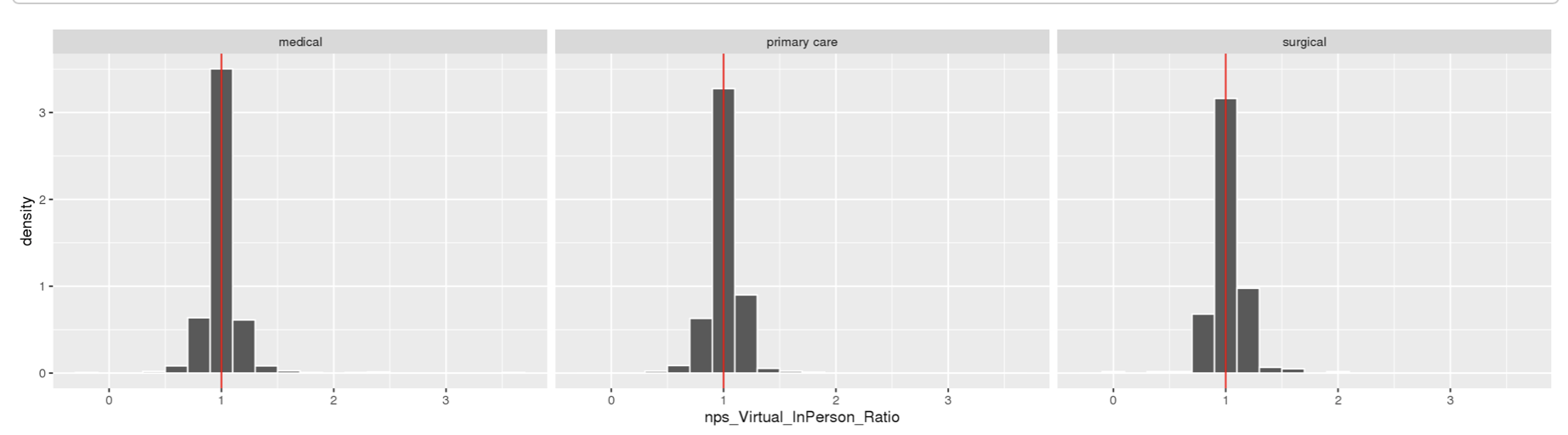 |
| --- | --- | --- |
